# Variation in Vegetation Phenology and Its Response to Climate Change in Marshes of Inner Mongolian

**DOI:** 10.3390/plants12112072

**Published:** 2023-05-23

**Authors:** Yiwen Liu, Xiangjin Shen, Jiaqi Zhang, Yanji Wang, Liyuan Wu, Rong Ma, Xianguo Lu, Ming Jiang

**Affiliations:** 1 Northeast Institute of Geography and Agroecology, Chinese Academy of Sciences, Changchun 130102, China; liuyiwen@iga.ac.cn (Y.L.); zjiaqizhang@126.com (J.Z.); wangyanji1@126.com (Y.W.); wuliyuan0@163.com (L.W.); maron40@yeah.net (R.M.); xianguoluv@163.com (X.L.); jiangmin71@163.com (M.J.); 2 University of Chinese Academy of Sciences, Beijing 100049, China

**Keywords:** marsh, vegetation phenology, climatic change, response, Inner Mongolia

## Abstract

Inner Mongolia has a large area of marsh wetland in China, and the marsh in this region is important for maintaining ecological balance. Understanding variations in vegetation phenology of marsh ecosystems and their responses to climatic change is crucial for vegetation conservation of marsh wetlands in Inner Mongolia. Using the climate and NDVI data during 2001–2020, we explored the spatiotemporal changes in the start (SOS), end (EOS), and length (LOS) of vegetation growing season and analyzed the effects of climate change on vegetation phenology in the Inner Mongolia marshes. Results showed that SOS significantly (*p* < 0.05) advanced by 0.50 days/year, EOS significantly delayed by 0.38 days/year, and thus LOS considerably increased by 0.88 days/year during 2001–2020 in marshes of Inner Mongolia. Warming temperatures in winter and spring could significantly (*p* < 0.05) advance the SOS, and increased summer and autumn temperatures could delay EOS in Inner Mongolia marshes. We found for the first time that daytime maximum temperature (T_max_) and night minimum temperature (T_min_) had asymmetric effects on marsh vegetation phenology. Increasing T_max_ had a stronger advancing effect on SOS than increasing T_min_ from December to April. The increase of T_min_ in August could obviously delayed EOS, while increasing T_max_ in August had no significant effect on EOS. This study highlights that the asymmetric influences of nighttime and daytime temperatures should be taken into account in simulating marsh vegetation phenology in temperate arid and semi-arid regions worldwide, particularly in the context of global asymmetric diurnal warming.

## 1. Introduction

As an important type of wetland ecosystem, marsh can significantly affect the global ecological environment and carbon cycle [1,2,3,4,5,6,7,8,9]. Vegetation is a crucial component of marshes and has a significant impact on ecosystem functions [10,11,12,13,14,15,16]. The vegetation phenology of marshes is sensitive to climate change [17,18,19]. In the context of climate change, the phenology of marsh vegetation has significantly changed [19,20,21,22]. Understanding the variations in vegetation phenology of marshes and their responses to climate change is vital for predicting regional ecosystem carbon exchange [18,23,24].

Inner Mongolia has a large area of marshes in China, and the marshes in this region significantly affect the biogeochemical cycle and biodiversity [25]. Previous researchers have studied the changes in vegetation phenology and their responses to climate variations in Inner Mongolia [26,27,28,29]. For example, Qiao and Wang [30] investigated the influence of climate change on the phenology of Inner Mongolia’s grasslands and discovered that increasing temperature and precipitation advanced the start date (SOS) of the vegetation growing season from 1982 to 2015. Sha et al. [31] studied the spatiotemporal variations in phenology and found that the increase of precipitation in the preseason remarkably advanced the SOS of grassland in Inner Mongolia during 1998–2012. Ren et al. [32] investigated the effects of climate change on grassland vegetation phenology. They found that the end date (EOS) of vegetation growing season for desert steppe was affected by preseason precipitation, while the EOS of meadows and temperate steppes was affected by the preseason air temperature in Inner Mongolia from 2000 to 2016 [32]. Wang et al. [28] analyzed the climatic influence on grassland phenology and suggested that increasing annual precipitation lengthened the length (LOS) of vegetation growing season for grassland in Inner Mongolia from 1982–2012. However, previous research mostly focused on grasslands, and studies on the vegetation phenology of marshes in Inner Mongolia are less. Compared with grassland ecosystems, marsh wetland ecosystems have distinctive environmental conditions, and the phenology of vegetation in marsh wetland ecosystems may respond differently to climate change [19,24,33]. To reveal the climate impact on marsh vegetation, analyzing the variations of vegetation phenology and their correlations with climate change in the marshes of Inner Mongolia is necessary. In addition, Shen et al. [34] suggested that nighttime and daytime warming had asymmetric influences on the SOS of temperate grasslands. The increase in daytime maximum temperature (T_max_) had a stronger advancing effect on SOS than increasing nighttime minimum temperature (T_min_) [34]. This may be because that T_min_ is more likely to be below the threshold temperature than T_max_ before the green-up of vegetation, so it contributes less to achieving the thermal requirement for green-up [34]. Marshes are wetter than grasslands [35]. Whether the impacts of diurnal temperatures on marsh vegetation phenology in Inner Mongolia are asymmetric is unclear. Given the background of asymmetric warming during the nighttime and daytime, it is necessary to explore the separate influence of T_min_ and T_max_ on vegetation phenology in the marshes of Inner Mongolia.

Using the climate and NDVI data during 2001–2020, we studied the spatiotemporal changes of SOS, EOS, and LOS in the marshes of Inner Mongolia. This study proposed two hypotheses: (1) Temperature may be the main factor affecting vegetation phenology in marsh wetlands of Inner Mongolia; (2) Daytime and nighttime temperatures may have different impacts on vegetation phenology in marsh wetlands of this region. Our aims were to explore the spatio-temporal variations of vegetation phenology and investigate the effects of climatic change, especially diurnal warming, on the vegetation phenology of marshes in Inner Mongolia. The results may provide important scientific references for predicting vegetation dynamics and carrying out adaptive management of marsh vegetation in this region.

## 2. Results

### 2.1. Changes in the Phenology of Marshes in Inner Mongolia from 2001 to 2020

**Figure 1:**
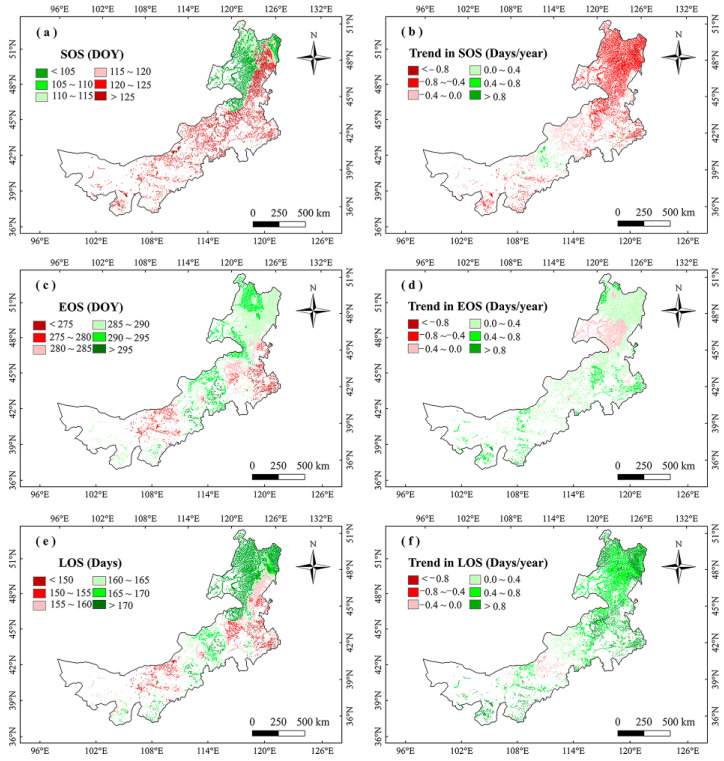
Distributions of the long-term mean (**a**,**c**,**e**) and change trends (**b**,**d**,**f**) of marsh vegetation SOS, EOS, and LOS in Inner Mongolia during 2001–2020.

From 2001 to 2020, the average vegetation SOS in the marsh of Inner Mongolia was approximately 122 days per year (DOY; May 2 or 3 for leap or non-leap years). The average EOS was approximately 280 DOY (October 7 or 8), and the average LOS was approximately 157 days. Spatially, the SOS was earlier in northeastern and later in southeastern and middle of Inner Mongolia (Figure 1a). The EOS in northeastern Inner Mongolia was later, while the earlier EOS was situated in southeastern Inner Mongolia (Figure 1c). The LOS was longer and shorter in the northeastern and southeastern regions, respectively (Figure 1e).

**Figure 2:**
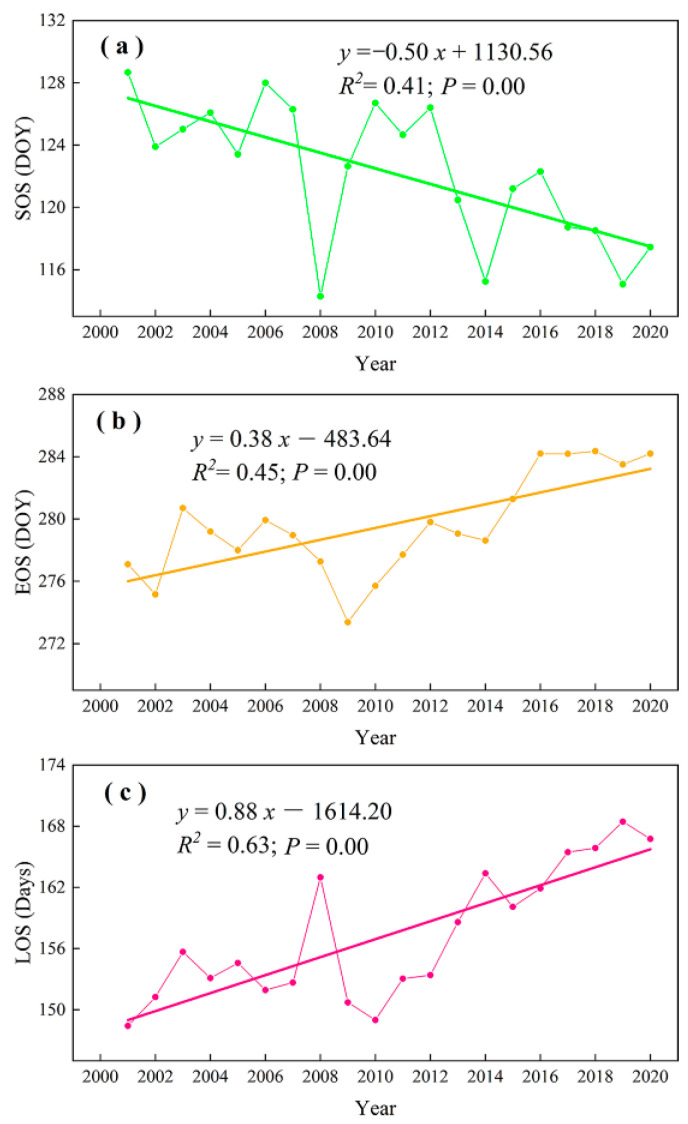
Temporal variations in SOS (**a**), EOS (**b**), and LOS (**c**) of marshes in Inner Mongolia during 2001–2020.

For the variations of vegetation phenology, SOS advanced significantly (*p* < 0.05) by 0.50 days/year, EOS delayed significantly by 0.38 days/year, and LOS increased remarkably by 0.88 days/year (Figure 2) in marshes of Inner Mongolia during 2001–2020. The larger advancing SOS trends mainly occurred in the east of the study region and the largest delaying trends were mainly situated in the middle regions (Figure 1b). The largest delaying and advancing trends of EOS were mainly distributed in the east and northeast regions, respectively (Figure 1d). In addition, the largest increasing and decreasing trends of LOS mainly occurred in the eastern and central regions, respectively (Figure 1f).

### 2.2. Correlations between Climatic Factors and Phenology in Marshes of Inner Mongolia from 2001 to 2020

**Figure 3:**
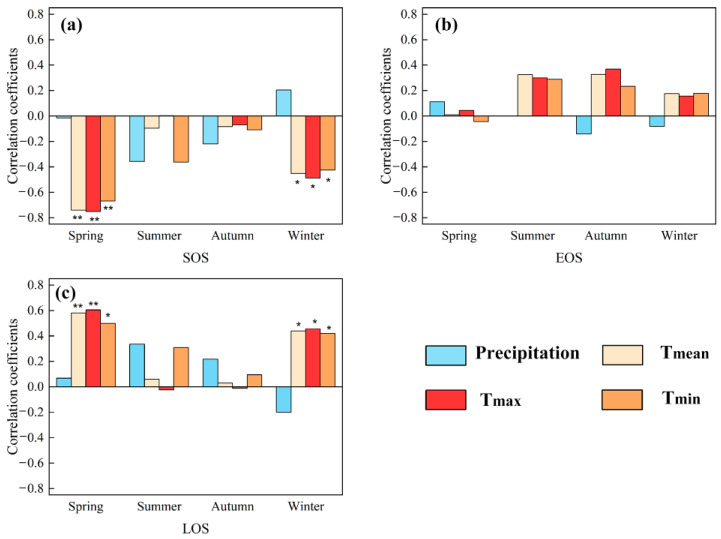
Correlations between vegetation SOS (**a**), EOS (**b**), LOS (**c**) and seasonal climate factors (precipitation, T_mean,_ T_max_, and T_min_) in marshes of Inner Mongolia during 2001–2020. ** and * mean significant at *p* < 0.01 and 0.05, respectively.

**Figure 4:**
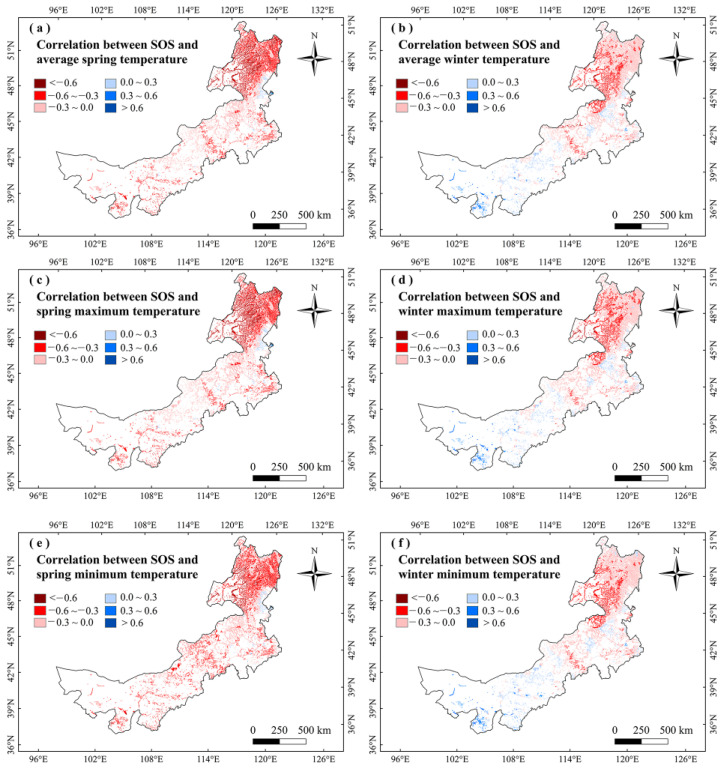
Relationships between SOS and temperatures (T_mean_, T_max_, and T_min_) in winter and spring in the marshes of Inner Mongolia during 2001–2020.

**Figure 5:**
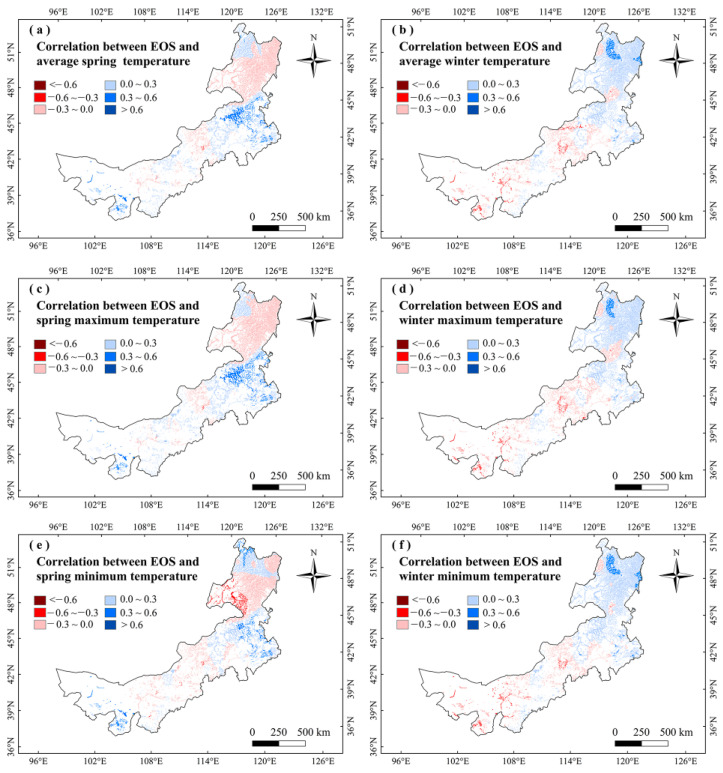
Relationships between EOS and temperatures (T_mean_, T_max_, and T_min_) in winter and spring in marshes of Inner Mongolia during 2001–2020.

**Figure 6:**
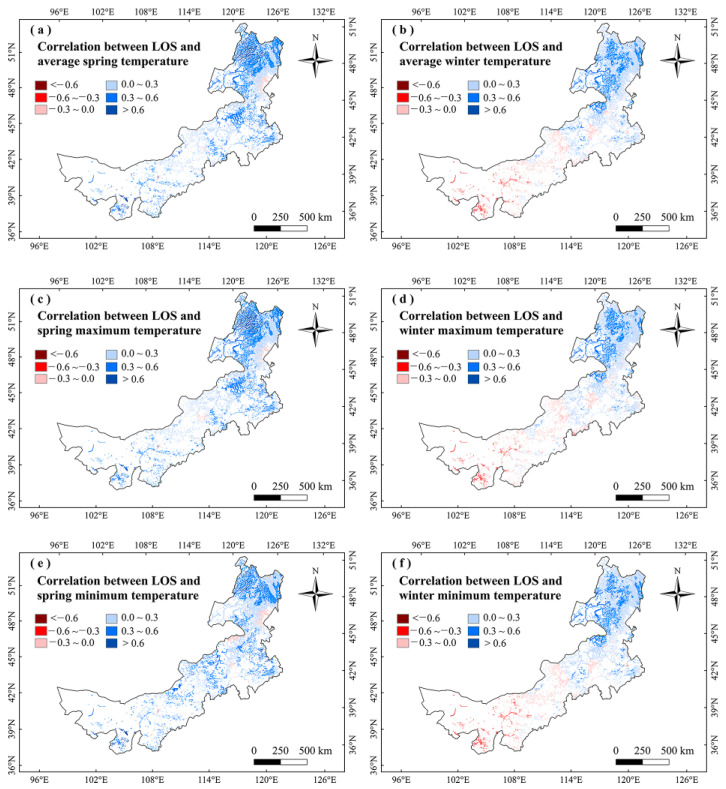
Relationships between LOS and temperatures (T_mean_, T_max_, and T_min_) in winter and spring in marshes of Inner Mongolia during 2001–2020.

For analyzing the climatic effects on marsh phenology, we explored the correlations between climate factors and vegetation phenology in Inner Mongolia marshes during 2001–2020. It showed no significant correlation between marsh phenology and precipitation in any season (Figure 3). The SOS had significant (*p* < 0.05) negative correlations with temperatures in winter and spring, and the largest negative correlations were mainly found in northeastern Inner Mongolia (Figure 3 and Figure 4). The correlations between EOS and temperature in autumn and summer were moderately positive, and the correlations between LOS and temperatures in winter and spring were significantly positive (Figure 3 and Figure 5). The LOS had the largest positive relationships with temperatures in winter and spring in northeastern Inner Mongolia, and the largest negative correlations were mostly situated in central and southwestern Inner Mongolia (Figure 6). The SOS and EOS had no significant correlations with precipitation in different months, whereas the correlation between LOS and precipitation in August was significantly positive (Table 1). The SOS was significantly negatively correlated with T_mean_ from December to April. The relationships between LOS and T_mean_ from January to March were significantly positive (Table 1). In terms of the influences of daytime and nighttime temperature on marsh vegetation phenology, SOS showed negative correlations with T_max_ and T_min_ from December to April, and the negative correlation with T_max_ was stronger than that with T_min_ in those months (Table 1). The relationship between EOS and T_min_ in August was significantly positive, while the relationship with T_max_ in this month was weakly positive (Table 1). The LOS had positive correlations with T_min_ and T_max_ from December to April, and the positive correlations with T_max_ in those months were stronger (Table 1). Moreover, the correlation between the LOS and T_min_ in August was obviously (*p* < 0.05) positive, while the correlation with T_max_ in August was negative (Table 1).

## 3. Discussion

### 3.1. Spatiotemporal Changes in Phenology in Marshes of Inner Mongolia during 2001–2020

We found that the vegetation SOS was earlier (Figure 1a), EOS was later (Figure 1c), and LOS was longer (Figure 1e) in marshes of eastern Inner Mongolia. Inner Mongolia has a monsoon climate, and the hydrothermal condition in the east was better than in the west [36], which possibly explains the later EOS, earlier SOS, and longer LOS in eastern Inner Mongolia. We found that the regional average SOS advanced, EOS delayed, and thus LOS increased during 2001–2020 in marshes of Inner Mongolia. This result was similar to Cui and Shi [37] who found that SOS advanced, EOS delayed, and LOS increased in most regions of Inner Mongolia. In northeastern Inner Mongolia, EOS and SOS advanced, while LOS increased in this region. It indicates that the advanced SOS may explain increased LOS in northeastern Inner Mongolia.

### 3.2. Correlations between Climate Factors and Marsh Vegetation Phenology

We found that the marsh vegetation phenology had no significant correlations with precipitation in any season (Figure 3). It indicates that seasonal precipitation may be not the major factor influencing the vegetation phenology in the marshes of Inner Mongolia. This finding is different from the results of Wang et al. [28] and Gong et al. [38], who found that precipitation was the main reason affecting phenology in the grasslands of Inner Mongolia. Grasslands are relatively dry and may need more water to maintain plant growth [39]. Therefore, precipitation is a major factor influencing grassland phenology [38]. Different from the grassland ecosystem, the marsh ecosystem has relatively humid and wetter than grasslands [40]. The water condition of marshes in this region is relatively sufficient. Therefore, precipitation in different seasons may not be the main factor affecting the phenology of marshes vegetation in this region.

For the impacts of climate change in different months on phenology, the relationships of SOS with T_mean_ from December to April were significant (Table 1). This suggests that increasing temperatures in winter and spring can advance SOS. The reason may be that the increase of temperatures in winter and spring can reduce frost [33], and promote heat accumulation to initiate green-up [41,42]. For climatic effects on EOS, our results showed that increasing temperatures in autumn and summer can delay EOS to a certain extent. This may be because increasing temperatures in summer and autumn promote photosynthetic enzyme activities and slow chlorophyll degradation during leaf senescence [43,44], thus delaying EOS.

For the responses of LOS to climate change, LOS has significant positive correlations with winter and spring temperatures (Figure 3). We thus conclude that increasing temperatures in spring and winter advanced SOS, thereby increasing LOS. In addition, LOS has an obvious (*p* < 0.05) positive correlation with precipitation in August, suggesting that increasing precipitation in August could increase the LOS (Table 1). In August, the climate is relatively dry (high temperature and strong evaporation) [45]. The vegetation in Inner Mongolia grows vigorously in August [46]. Increasing precipitation in August may alleviate water stress and promote the growth of marsh vegetation [43,47]. This may partly explain the increasing inprecipitation in August and the LOS increases in the marsh of Inner Mongolia.

In terms of the influences of T_min_ and T_max_ on marsh vegetation phenology, there was an asymmetric impact of T_min_ and T_max_ on vegetation phenology in marshes of Inner Mongolia. The increases of T_min_ and T_max_ in winter and spring advanced SOS, while increases of T_max_ in winter and spring had a stronger advancing effect on the SOS than T_min_ (Figure 3). This is because the climate in Inner Mongolia is cold in winter and spring [36], and vegetation needs heat accumulation to start growing [48,49]. Increasing T_max_ and T_min_ in winter and spring may promote heat accumulation and reduce frost damage [19,41,50]. Before the green-up of vegetation, the T_min_ is more likely to be below the threshold of heat accumulation, thus T_min_ may have a weaker influence on the green-up of plants than T_max_ [34,50]. This may explain why the increase of T_max_ in winter and spring had a stronger advancing effect on the SOS than T_min_ in the marshes of Inner Mongolia.

We found that the correlation between the EOS and T_min_ in August was obviously positive, while the correlation with T_max_ in August was weakly positive (Table 1). This implied that the increases of T_min_ and T_max_ in August delayed the marshes EOS in Inner Mongolia, but increasing T_min_ had a stronger delaying effect on the EOS than that in T_max_. The delaying effect of warming T_max_ on EOS may be due to the fact that the increase in T_max_ can improve the activities of photosynthetic enzymes and promote photosynthesis [51]. By contrast, although the increase of T_min_ can cause more organic matter consumption, increasing T_min_ can also cause a compensation effect for marsh vegetation [52]. The compensation effect is the phenomenon where the increases in vegetation respiration and organic matter consumption due to warming temperature at night are compensated by enhanced photosynthesis the next day [35,53,54]. The compensation effect is stronger in regions with suitable hydrothermal conditions, and can even result in overcompensation [55,56,57]. This compensation effect may produce a state in which photosynthesis restores or exceeds respiration [58]. In our study, the water and nutrient status of marshes in Inner Mongolia was conducive to the overcompensation effect in the vegetation. The most suitable water and nutrient conditions occurred in August, and August is the best time to cause an overcompensation effect in the vegetation [59]. Therefore, the increased T_min_ in August may cause an overcompensation effect for marsh vegetation, which explains stronger delaying effects on EOS than T_max_ in marshes of Inner Mongolia.

Our results showed that LOS had a stronger positive correlation with T_max_ than with T_min_ from December to April (Table 1). It indicates that the increases in T_min_ and T_max_ in winter and spring increase LOS, and rising T_max_ in winter and spring had stronger effects on LOS than T_min_ in marshes of Inner Mongolia. On one hand, increasing T_max_ and T_min_ in winter and spring may promote heat accumulation and reduce frost damage [41,50]. On the other hand, the T_min_ is more likely to be below the threshold of heat accumulation before the green-up of vegetation, thus T_min_ may have a weaker influence on the green-up of plants than T_max_ [50]. The increases of T_min_ and T_max_ in winter and spring might advance SOS, thereby increasing the vegetation n LOS in marshes of Inner Mongolia. In addition, LOS was significantly positively correlated with T_min_ in August (Table 1), confirming that warming T_min_ in August can delay EOS and thus increase LOS. It owing to the fact that August is the best time to occur an overcompensation effect in the vegetation [60]. Therefore, the increased T_min_ in August may cause an overcompensation effect for marsh vegetation. This may explain the delayed EOS and thus increased LOS.

**Figure 7:**
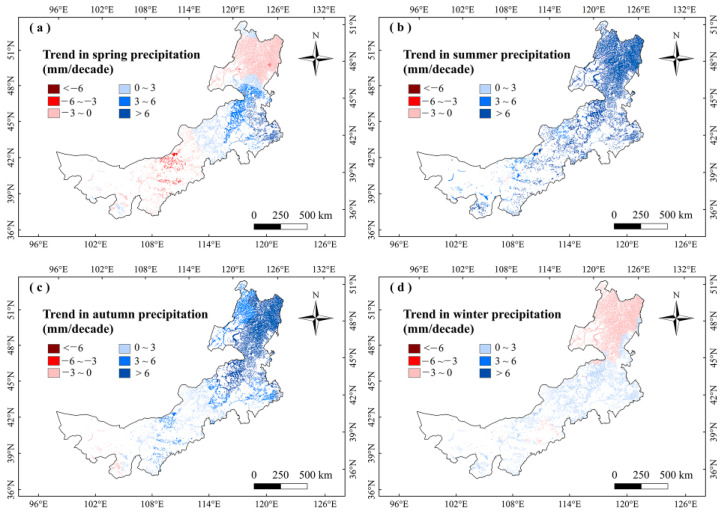
Change trends of precipitation (mm/decade) in different seasons in marshes of Inner Mongolia during 2001–2020.

**Figure 8:**
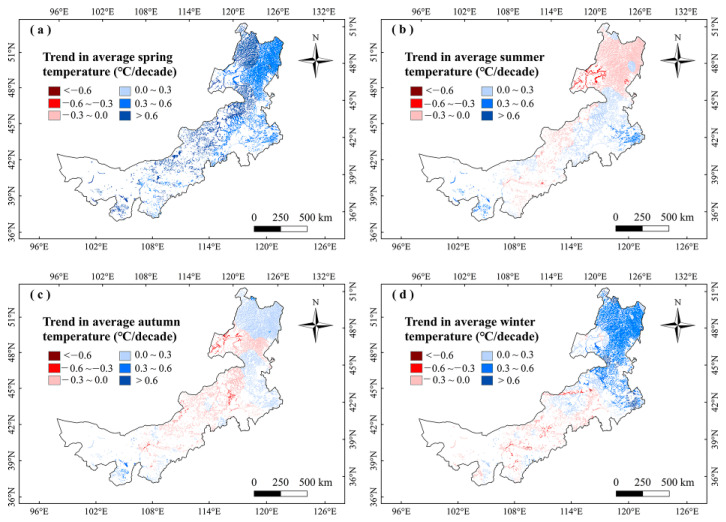
Change trends of average temperatures (°C/decade) in different seasons in marshes of Inner Mongolia during 2001–2020.

**Figure 9:**
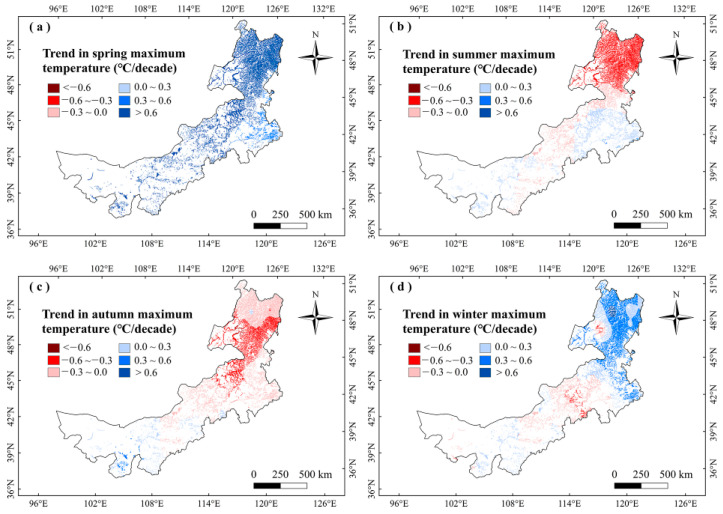
Change trends of maximum temperatures (°C/decade) in different seasons in the marshes of Inner Mongolia during 2001–2020.

**Figure 10:**
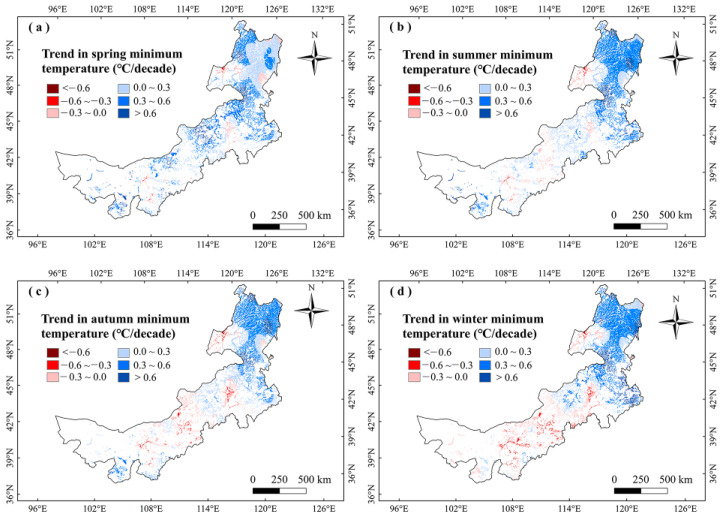
Change trends of minimum temperatures (°C/decade) in different seasons in marshes of Inner Mongolia during 2001–2020.

To further explain the climate impacts on vegetation phenology, we explored the changes in climate factors in Inner Mongolia marshes during 2001–2020 (Figure 7, Figure 8, Figure 9 and Figure 10). We found that precipitation in summer and autumn significantly increased, while it showed weak variations in spring and winter during this period (Table 2). The temperatures increased in different seasons. The T_max_ increased in spring and winter, while it decreased in summer and autumn. The T_min_ moderately increased in different seasons (Table 2). The correlations between LOS and precipitation in summer and autumn were moderately positive in this region (Figure 3), indicating that increasing precipitation in summer and autumn may explain the increase in LOS of marsh vegetation to some extent. Considering that SOS (LOS) showed a negative (positive) relationship with temperatures in winter and spring (Figure 3), we conclude that the increases in temperatures in winter and spring could explain the advanced SOS and increased LOS of Inner Mongolia marshes. In different months, the precipitation and T_min_ in August showed a significant increasing trend. Because LOS was significantly positively correlated with precipitation and T_min_ in August (Table 1), the increases in precipitation and T_min_ in August could explain the increase of LOS in the marsh of Inner Mongolia. Spatially, we found that the largest increasing trends in temperatures in winter and spring occurred in northeast Inner Mongolia (Figure 8a,d). The SOS (LOS) showed negative (positive) relationships with temperatures in winter and spring in this region (Figure 4 and Figure 6). Therefore, we inferred that increasing temperatures in spring and winter could explain the advanced SOS and increased LOS in northeastern Inner Mongolia.

### 3.3. Limitations

We should note that the study may have some uncertainties and limitations. Firstly, the NDVI data used in this work may have some uncertainties due to the influences of solar altitude angle and clouds. Secondly, we extracted unchanged marsh vegetation as the research area, but it cannot completely exclude the impacts of human activities. Therefore, in future research, it is necessary to further investigate the impacts of human activities on marsh vegetation in this region. Thirdly, the number of weather stations was limited in the study area, which perhaps affects the results. More accurate climate and vegetation index data are still needed to further confirm our results. Moreover, the current study only investigated the responses of vegetation phenology to temperature and precipitation. Human activities and other climatic factors can also influence the marshes vegetation in Inner Mongolia. As such, further research needs to explore the influences of other environmental and climatic variables on the vegetation phenology of marshes in Inner Mongolia.

## 4. Materials and Methods

### 4.1. Study Area

**Figure 11:**
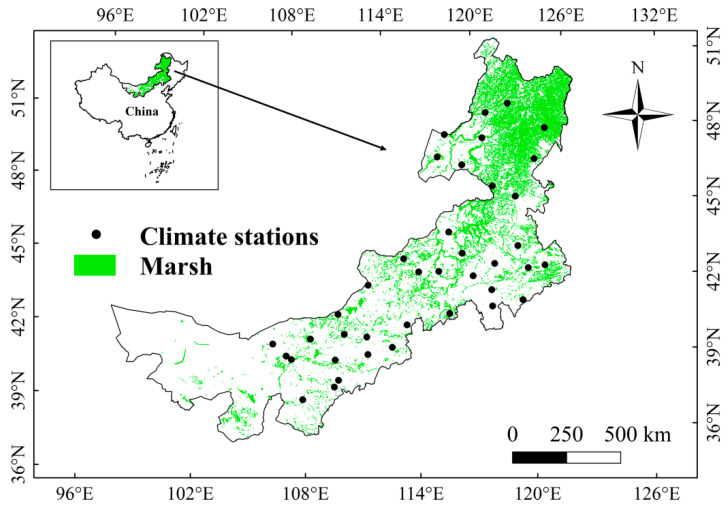
Spatial distributions of marshes and climate stations in Inner Mongolia.

Inner Mongolia is situated in the north of China (Figure 11). This region has a monsoon climate: dry and cold in winter, and wet and warm in summer [36,61,62,63]. Inner Mongolia is an important marsh distribution region in China, and the marsh in this region is important for the biogeochemical cycle and biodiversity conservation [25]. The marsh vegetation in Inner Mongolia mainly includes *Carex lasiocarpa*, *Carex pseudocuraica*, and *Betula fruticosa* [19]. 

### 4.2. Data

We used monthly precipitation, minimum, maximum, and average temperatures (T_mean_) data from 39 weather stations in Inner Mongolia during 2001–2020 in this study (Figure 1). The climate data were provided by China Meteorological Center, and they have been subjected to strict quality assurance [33]. This study also used the MOD13Q1 NDVI dataset from 2001 to 2020, obtained from NASA. The spatial-temporal resolutions of NDVI data were 250 m and 16 days, respectively [52]. The distribution data of marshes in 2015 and 2000 covering Inner Mongolia were provided by the Earth System Science Data Center of China and passed through strict quality control and verifications [64]. The spatial resolution of marsh distribution data was 30 m [64].

### 4.3. Method

To avoid the possible impacts of land use change on phenological results, we extracted the unchanged marshes (marshes in both marsh distribution maps) as the study region. To calculate the SOS and EOS in marshes of Inner Mongolia, we used the Polyfit-Maximum approach, which is widely applied in previous research because of its good performance [19,24,34,65,66,67]. Firstly, we calculated the changes in NDVI by the following formula:

(1) $$NDVIrate(t)=\frac{NDVI(t+1)-NDVI(t)}{NDVI(t)}$$

NDVI(t) refers to NDVI change, and t is the Julian date (DOY). This method set the largest decrease (increase) date of NDVI to the corresponding end (start) date of the growing season. We used the EOS and SOS to calculate the LOS of marsh vegetation in Inner Mongolia.

Then, this study applied the 6-degree polynomial function to better fit the NDVI time series [60]. Its formula is as follows:

NDVI = a_0_ + a_1_d + a_2_d^2^ + … + a_6_d^6^ (2)

where a_1_, …a_6_ refer to the fitting coefficients of the least square regression.

This study adopted the ordinary Kriging approach to interpolate climate data into the distribution of Inner Mongolia marshes and then unified the climate and NDVI dataset to the same spatial resolution [19,48]. We used monthly climate data to calculate the average values of precipitation and temperature in spring (March–May), summer (June–August), autumn (September–November), and winter (previous December–February). The average value of all pixels for each variable was used to calculate the regional average value of marshes in this region. In addition, we calculated the variation trends in the variables by linear regression analysis as follows [40]:

(3) $$\theta_{slope}=\frac{\left(n\times \sum_{i=1}^{n}i\times Mi\right)-\left(\sum_{i=1}^{n}i\times \sum_{i=1}^{n}Mi\right)}{n\times \sum_{i=1}^{n}i^{2}-{\left(\sum_{i=1}^{n}i\right)}^{2}}$$

*M*_i_ is the climatic factors (or phenology) value in the *i* th year; *i* is the serial year number; n represents the study period; *θ*_slope_ is the change slope in climatic factors (or phenology) of each pixel, *θ*_slope_ < 0 represents that the variable reduced, otherwise it increased.

Using Pearson correlation analysis, we explored the correlation between phenology and climatic factors in different months [52].


(4) $$R_{ab}=\frac{\sum_{i=1}^{n}\left(a_{i}-\bar{a}\right)\left(b_{i}-\bar{b}\right)}{\sqrt{\sum_{i=1}^{n}{\left(a_{i}-\bar{a}\right)}^{2}}\sqrt{\sum_{i=1}^{n}{\left(b_{i}-\bar{b}\right)}^{2}}}$$

*n* represents the length of the period; *R*_*ab*_ refers to the correlation coefficient; *a_i_* and *b_i_* are the average climatic variables and phenology in year *i*; $\overset{-}{a}$ and $\overset{-}{b}$ refer to the average climate and phenology variables from 2001–2020.

## 5. Conclusions

From 2001 to 2020, the SOS advanced significantly (*p* < 0.05) by 0.50 days/year, the EOS significantly by 0.38 days/year, and the LOS increased obviously by 0.88 days/year in the marshes of Inner Mongolia. The increases in temperature in winter and spring could significantly advance the SOS, thus increasing the LOS in Inner Mongolia marshes. Increasing temperatures in autumn and summer delayed the EOS to a certain extent. In different months, increasing precipitation in August could obviously increase the LOS, and increased temperatures from December to April could advance SOS. Furthermore, we found for the first time that T_max_ and night T_min_ had asymmetric effects on phenology. Increasing T_max_ had a stronger advancing effect on SOS than increasing T_min_ from December to April. The increase of T_min_ in August obviously delayed EOS, while increasing T_max_ in August had no significant effect on EOS. This study highlights the diverse impacts of monthly precipitation and temperature changes on marsh vegetation phenology and implies that the asymmetric influences of nighttime and daytime temperatures should be taken into account when simulating marsh vegetation phenology in temperate arid and semi-arid regions worldwide, particularly in the context of global asymmetric diurnal warming. The results of this study can contribute to predicting vegetation dynamics of marshes and provide an important scientific basis for the conservation of marsh vegetation in Inner Mongolia.

## Figures and Tables

**Table 1:** Correlation coefficients between vegetation phenology and climate variables (precipitation, T_mean_, T_max_, and T_min_) in marshes of Inner Mongolia during 2001–2020.

	SOS				EOS				LOS			
	Precipitation	T_mean_	T_max_	T_min_	Precipitation	T_mean_	T_max_	T_min_	Precipitation	T_mean_	T_max_	T_min_
January	−0.029	−0.422 *	−0.443 *	−0.387	−0.021	0.259	0.238	0.276	0.012	0.458 *	0.464 *	0.439 *
February	−0.083	−0.272	−0.328	−0.251	0.053	0.133	0.105	0.140	0.091	0.278	0.307	0.265
March	0.338	−0.691 **	−0.734 **	−0.618 **	−0.121	0.139	0.152	0.100	−0.323	0.606 **	0.646 **	0.530 *
April	0.202	−0.530 *	−0.567 **	−0.363	−0.192	−0.050	−0.025	−0.134	−0.253	0.387	0.428 *	0.215
May	−0.207	−0.081	−0.072	−0.141	0.247	−0.187	−0.099	−0.206	0.285	−0.031	0.006	0.006
June	−0.185	0.113	0.149	−0.070	−0.313	0.217	0.284	−0.046	−0.017	−0.039	−0.006	−0.118
July	−0.190	−0.308	−0.333	−0.147	0.004	0.272	0.275	0.141	0.010	0.324	0.327	0.226
August	−0.332	−0.134	0.068	−0.305	0.218	0.197	0.036	0.454 *	0.566 **	−0.097	−0.328	0.486 *
September	−0.277	−0.065	0.056	−0.183	−0.056	0.408	0.298	0.300	0.220	0.212	0.038	0.321
October	−0.168	0.192	0.109	0.224	−0.203	0.245	0.317	0.093	0.290	−0.058	−0.058	−0.068
November	0.310	−0.220	−0.235	−0.179	0.136	−0.104	−0.088	−0.118	−0.173	0.119	0.138	0.080
December	0.332	−0.459 *	−0.483*	−0.446 *	−0.120	0.011	0.025	−0.013	−0.318	0.362	0.388	0.340

** and * mean significant at *p* < 0.01 and 0.05, respectively.

**Table 2:** Change trends of seasonal and monthly precipitation (mm/year), T_mean_ (°C/year), T_max_ (°C/year), and T_min_ (°C/year) in marshes of Inner Mongolia during 2001–2020.

	Precipitation	T_mean_	T_max_	T_min_
Spring	0.058	0.060	0.083	0.030
Summer	1.527 *	0.047	−0.024	0.034
Autumn	0.788 *	0.002	−0.023	0.027
Winter	−0.001	0.031	0.032	0.028
January	−0.055	0.038	0.046	0.032
February	0.089	0.006	0.005	0.006
March	−0.151	0.098	0.123	0.066
April	−0.434	0.039	0.075	−0.018
May	0.757	0.042	0.051	0.042
June	0.750	−0.040	−0.059	−0.009
July	0.590	0.042	0.054	0.035
August	3.240 **	−0.010	−0.067	0.078 **
September	1.990 *	0.011	−0.038	0.072 *
October	0.266	−0.017	−0.018	−0.013
November	0.109	0.012	−0.012	0.023
December	−0.093	0.011	0.012	0.008

** and * mean significant at *p* < 0.01 and 0.05, respectively.

## Data Availability

The data used in this article can be requested by the author.
